# Dr. Koch’s Treatment for Tuberculosis

**Published:** 1890-12

**Authors:** 


### EDITORIAL DEPARTMENT.

### DR. KOCH'S TREATMENT FOR TUBERCULOSIS.

In an address before the International Medical Congress, last Summer, Dr. Koch said: "For years past I have been seeking means for the therapeutic treatment of consumption, but I began with pure cultivation of the bacillus. I found a number of substances—ethereal oils, tar pigments, mercurial vapor, salts of gold and silver, especially cyanide of gold—some of which, like the last, even when strongly diluted, prevent the growth of the bacillus, which, of course, suffices to bring the disease to a standstill. All these substances, however, have proved ineffectual when used against the bacillus in the bodies of animals. I continued my search, however, and found what I sought."

But the discovery was not sufficiently developed to be placed before the public at that time. Six weeks ago the world was startled by the announcement that a remedy had been found, and was being used in the treatment of human beings suffering with tuberculosis.

Dr. Koch states: "There is very little use of my saying just now what the inoculating fluid is or how I obtained it. It is a lymph, a brownish, transparent liquid. It is so prepared as to be proof against deterioration. When, however, it is diluted with water to the necessary degree for use, the matter is liable to decay. It is necessary, therefore, that the attenuations should be perfectly sterilized by heat and preserved in wadding covering, or prepared with a solution of phenol 50 per cent. strong. It has cost me years of my life, and I propose to retain the secret for a few weeks longer from publicity, though it is already known to my assistants and to many of my professional friends. Its preparation demands infinite pains and exactness, and it is being prepared by my assistant, Dr. Libbertz, to whom I have confided this important part of my work. I believe I am discreet on this subject with good and sufficient reason. The experience of premature disclosures has made me wise. I calculate I have wasted one year of my life in combating some captious and not perfectly conscientious critics of my original work. Were I to publish how the first stage of the discovery was made, the exact ingredients and the method of the preparation of the fluid, thousands of medical men from Moscow to Buenos Ayres would to-morrow be engaged in concocting it, and injecting it, too, for that matter. I think I am right in supposing, as I do, that more than half these gentlemen are incompetent to prepare the fluid which, with special study and special opportunities, it has taken me a year to prepare. Then these experiments might cause incalculable harm to thousands of innocent patients, and, at the same time, bring into discredit a system of treatment which, I believe, will prove a boon to all mankind. If experiments now being made turn out successfully, then the medical world will find me and my devoted assistants only too ready to initiate them into the intricacies of the treatment without the least reserve. But until then, although it seems, perhaps, selfish, I really claim it as at once our duty and the purest unselfishness. They must content themselves with being patient. In the meantime I advise them to be very chary as to the statements which appear in the press regarding our progress."

When taken into the stomach, the curative matter proves to have no effect. It must be applied subcutaneously. The kind of syringe recommended by Professor Koch is one furnished with a small, hollow rubber ball. Twenty-five-hundredths of a cubic centimeter intensely affected a healthy, full-grown man who was subjected to experiment.

Prof. Koch experimented with the fluid upon his own body, and describes the effect. He injected twenty-five hundredths of a cubic centimeter of the fluid under the skin of his upper arm. Three or four hours after the injection was made he experienced a contraction of the limbs and a marked feeling of lassitude. At the same time he felt a desire to cough, together with difficulty of breathing. These symptoms increased rapidly, and in the fifth hour he experienced an unusually violent rigor. The shivering lasted for nearly an hour, and was accompanied with nausea and vomiting. The temperature of his body rose to 39.6° Centigrade. After a period of twelve hours the symptoms began to abate, the temperature of the body declined, and on the following day resumed its normal degree. The heaviness of the limbs and the feeling of lassitude, however, continued for some days, during which time the point on his arm at which the injection was made continued to be painful and remained red.

In a case like this, when such statements come from a scientific man of the ability, reputation and record of Dr. Koch, we can but accept the fact that he has arrived at great results. But we must regard his reticence and secrecy as an acknowledgment that the work is not yet entirely satisfactory to himself; that he has been forced for some reason, best known to himself, to bring his subject before the world in a somewhat more crude condition than that in which he gave his other discoveries to the public. Believing such to be the case, we consider that Dr. Koch is perfectly wise and justified in retaining his secret until it has been matured and sufficiently developed to be handled by others without danger. It remains, however, a problem that the first experiments should have been made on human beings, when the bovine race offers so numerous and inexpensive subjects which might have been treated and destroyed at will to show the results obtained.

He says: To sum up the results of the treatment so far in the cases in which the physical condition of the patient was good—Lupus has yielded easily to the inoculating treatment, even when the cases have been of many years' standing. The bacilli have been destroyed completely after a number of injections, of course varying with each case, and the web of lupus has in some cases been sloughed off, but in the majority it has been easily removed surgically. The narbe or star which marks the spot where it existed is not so large or the disfigurement anything like so great as is the case with the sharp spoon treatment, in which the tissue is scooped out. Consequently the danger of lessening the usefulness of the affected limb is much less, and I think the danger of a recurrence is also lessened.

"As to the effect upon living tuberculosis, though the disclosure is premature, I will tell just how we stand. I have twenty patients with whom I personally follow the treatment minutely, and they represent (and I have, of course, chosen them on this account) the graduated stages of the disease. In fifteen of these patients the bacilli have completely disappeared from the sputa. They have gained much in weight, in general appearance, and in spirits, which last is not to be a neglected symptom. In the remaining five cases I regret to say there is not the slightest indication that the ordinary course of the disease has been stopped. These are cases in which I found already large cavities in the lungs. In these the cough, the rattle in the throat, and the almost undiminished number of bacilli in the sputa continues. All of these symptoms, I repeat, have disappeared in the other cases. Of course, nothing can be considered final yet, the first injection to a human being having only been made seventy days ago. I hope for good results in all cases in which the vital organs are intact."

